# Plasmid Sequence and Availability for an Improved Clostridioides difficile CRISPR-Cas9 Mutagenesis System

**DOI:** 10.1128/mra.00833-22

**Published:** 2022-11-07

**Authors:** Joshua N. Brehm, Joseph A. Sorg

**Affiliations:** a Department of Biology, Texas A&M University, College Station, Texas, USA; Indiana University, Bloomington

## Abstract

A two-plasmid mutagenesis system for Clostridioides difficile is described that improves ease of use and efficiency in creating site-directed mutations. pJB06 contains a xylose-inducible *cas9* gene, while the second plasmid (pJB07) encodes the corresponding guide RNA (gRNA) and regions of homology for repair of the introduced double-stranded DNA (dsDNA) breaks, both of which are replaceable via restriction digest.

## ANNOUNCEMENT

Developing a reliable and versatile method of generating targeted mutations in Clostridioides difficile is important to further our understanding of its pathogenesis. We previously designed a single-plasmid CRISPR-Cas9 system that uses a type II-A CRISPR system that was codon optimized for expression in C. difficile (1). Here, we seek to improve on that system.

CRISPR-Cas9 mutagenesis works through the combined effects of a Cas9-mediated double-stranded DNA (dsDNA) break and the endogenous homologous recombination system. This dsDNA break is introduced by Cas9 at a locus matching a 20-bp guide RNA (gRNA) sequence. The specificity of chromosomal cleavage is further increased by the requirement of the downstream protospacer adjacent motif (PAM) sequence 5′-NGG-3′ (2). The introduced dsDNA break is then repaired by recombination with the regions of homology, resulting in the introduction of the desired mutation (e.g., deletion) (3, 4).

**FIG 1:**
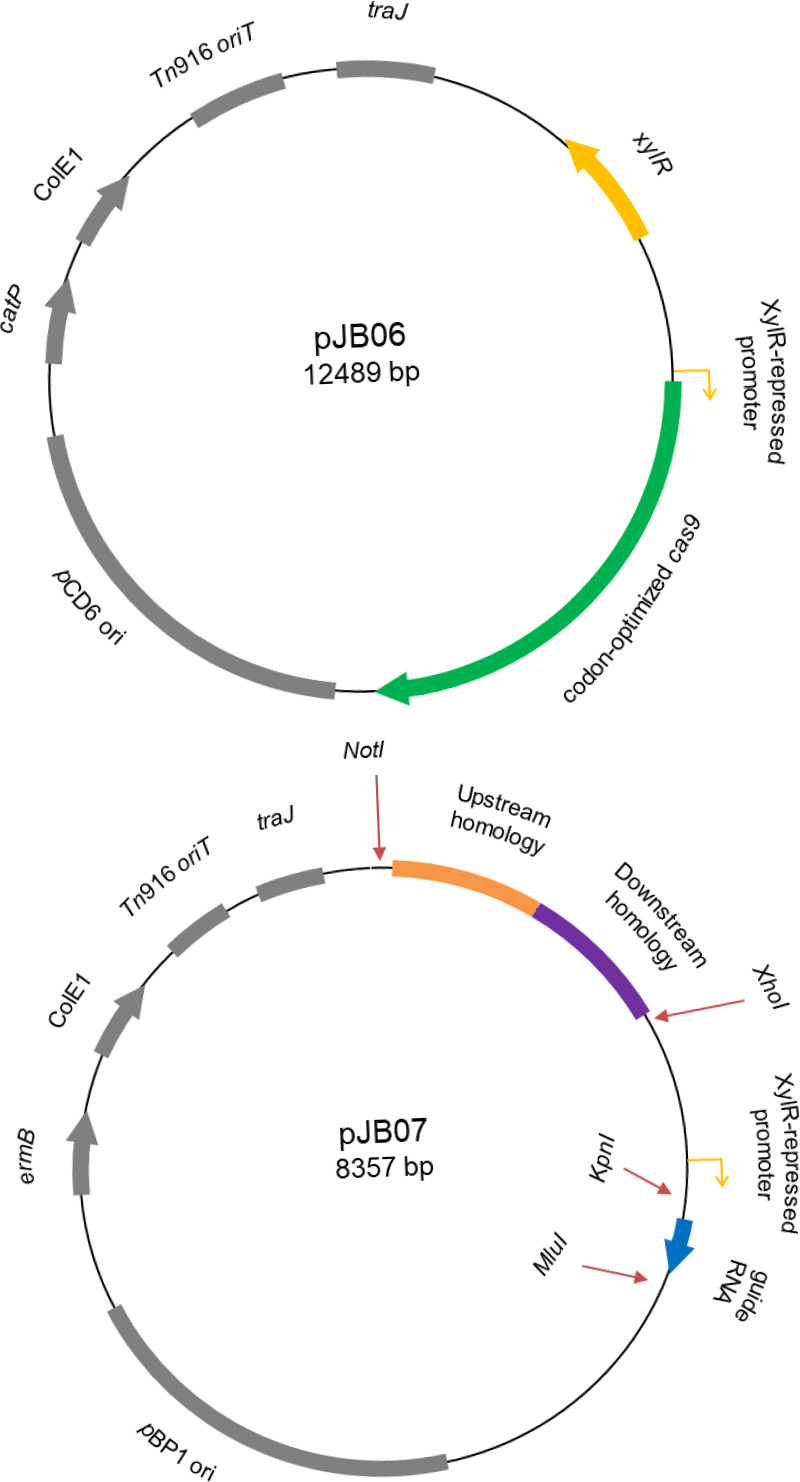
Maps of the pJB06 (*cas9*-containing) and pJB07 (homology- and gRNA-encoding) plasmids. The pJB06 and pJB07 plasmids have been engineered to carry both the Tn*916 oriT* and *traJ* regions for conjugal transfer from B. subtilis or E. coli, respectively. Both the *cas9* gene and the gRNA are under the conditional expression of a xylose-inducible promoter. The pJB06 plasmid is maintained in a strain of interest, and modified pJB07 plasmids are introduced to generate the desired mutations.

In the prior system, we observed that some mutagenesis plasmids never created the desired mutation, while others could never be introduced into C. difficile. We hypothesized that this could be due to toxicity of the plasmid when both *cas9* and the gRNA were located on the same plasmid. Therefore, we split the system into two separate plasmids (Fig. 1). pJB06 contains a XylR-repressed (5) *cas9* gene and is the base plasmid for the two-plasmid system. It can be stably maintained, with selection, and stored for future use.

The second plasmid, pJB07, contains two modular regions designed to be removed by restriction digest and replaced through Gibson assembly or standard ligations. The first module contains regions of homology flanking the desired insertion (if any) to be used in repairing the host chromosome. The second contains a sequence encoding a gRNA. Once transcribed, the gRNA directs the Cas9 endonuclease to the specified chromosomal region for cleavage. In addition, the promoter driving gRNA expression has been changed from the constitutively active, *gdh* promoter to the same xylose-inducible promoter used for *cas9* expression. This change should allow for simultaneous, conditional expression, while also reducing premature chromosomal cleavage due to excess gRNA production and increasing the efficiency of conjugations. Both plasmids also now contain two origins of transfer: *traJ* for conjugal transfer from Escherichia coli (6), and the Tn*916 oriT* for conjugal transfer from Tn*916*-containing Bacillus subtilis (6).

### Plasmid assembly.

pJB06 was constructed as follows. P*xylR*-controlled, codon-optimized *cas9* genes were amplified using primers 5′PxylR_COcas9 and 3′PxylR_COcas9 (Table 1) and inserted via Gibson assembly into pJS116 digested with NotI/XhoI, yielding pJB10. Both sequences for conjugal transfer were amplified using primers 5′Tn916_ori_gibson and 3′traJ and inserted via Gibson assembly into pJB10 digested with ApaI, yielding pJB06.

**TABLE 1:** Primers and gBlocks used in the construction of pJB06 and pJB07

Primer name	Primer sequence
5′PxylR_COcas9	TTATCAGGAAACAGCTATGACCGCGGCCGCCCCTTATATTTCATTAATTAAAGTTAAATT
3′PxylR_COcas9	TGCCAAGCTTGCATGTCTGCAGGCCTCGAGTTAATCACCACCTAATTGAGATAAATC
5′Tn916_ori_Gibson	CGGAAGAGCGCCCAATACGCAGGGCCCTAACATCTTCTATTTTTCCCAAATC
3′traJ	AATTTATCTACAATTTTTTTATCCTGCAGGGGGCCCGATCGGTCTTGCCTTG
5′pyrE_UP	TTATCAGGAAACAGCTATGACCGCGGCCGCGACGTGATTTTTAATGGGTA
3′pyrE_DN	TGCCAAGCTTGCATGTCTGCAGGCCTCGAGAAGCATTGATGTTCTTCCTTC
pyrE_gRNA_PxylR	GCCCTTCCCAACAGTTGCGCAGCCTGAATGGCGAATGGCGCTAGCTGACAAAGATAATTAAATATTTTATTATTAGTTCATAAGTTAGTTTAATATACTAACAAAAATAAAGCAAGTAAAATATACCTAAAATATAAAAAAATTAGGATAGGAAAACGATAGTTATGAAGTGGCATTCAAGGAGGGGGTACCGAAAAGTGATGCATTGTTGGGTTTTAGAGCTAGAAATAGCAAGTTAAAATAAGGCTAGTCCGTTATCAACTTGAAAAAGTGGCACCGAGTCGGTGCTTTTTTTCTATGGAGAAATCTAGATCAGCATGATGTCTGACTAGACGCGTGCTAGCATAAAAATAAGAAGCCTGCATTT

pJB07 was constructed as follows. Regions of homology were amplified with primers 5′pyrE_UP and 3′pyrE_DN and then inserted via Gibson assembly into pMTL82254 digested with NotI/XhoI. The resulting plasmid was digested with NheI, and gBlock pyrE_gRNA_PxylR containing P*xylR*-controlled gRNA sequence targeted to C. difficile *pyrE* was inserted via Gibson assembly, resulting in pJB11. pJB11 was then digested with ApaI, and the fragment required for both conjugal transfer systems (primers 5′Tn916_ori_gibson and 3′traJ) was inserted via Gibson assembly, resulting in pJB07.

### Data availability.

The complete sequences and availability of both plasmids are obtainable through the Addgene depository at https://www.addgene.org/depositing/81505/ (pJB06 ID 190480 and pJB07 ID 190481).
